# Multi-allelic positional Burrows-Wheeler transform

**DOI:** 10.1186/s12859-019-2821-6

**Published:** 2019-06-06

**Authors:** Ardalan Naseri, Degui Zhi, Shaojie Zhang

**Affiliations:** 1 Department of Computer Science, University of Central Florida, Orlando, FL, 32816 USA; 2 School of Biomedical Informatics, University of Texas Health Science Center at Houston, Houston, TX, 77030 USA

**Keywords:** PBWT, Multi-allelic, Haplotype matching

## Abstract

**Background:**

Recent advances in whole-genome sequencing and SNP array technology have led to the generation of a large amount of genotype data. Large volumes of genotype data will require faster and more efficient methods for storing and searching the data. Positional Burrows-Wheeler Transform (PBWT) provides an appropriate data structure for bi-allelic data. With the increasing sample sizes, more multi-allelic sites are expected to be observed. Hence, there is a necessity to handle multi-allelic genotype data.

**Results:**

In this paper, we introduce a multi-allelic version of the Positional Burrows-Wheeler Transform (mPBWT) based on the bi-allelic version for compression and searching. The time-complexity for constructing the data structure and searching within a panel containing t-allelic sites increases by a factor of *t*.

**Conclusion:**

Considering the small value for the possible alleles *t*, the time increase for the multi-allelic PBWT will be negligible and comparable to the bi-allelic version of PBWT.

## Background

The enormous amount of genotype data generated by the whole-genome sequencing or SNP arrays present a challenge to store and analyze them. Detection of large consecutive matches in a panel of genotype data is of great interest. A genotype panel is comprised of a set of alleles for multiple individuals. The long matches may represent Identical by Descent (IBD) segments which are the identical segments that have been passed by a common ancestor. IBD detection has a wide range of applications in genetics [1].

Genotype data in diploid organisms include the genetic information from both parents. Haplotype sequences contain the genotype information and the sequences from each parent have been separated. The naive approach for finding exact matches in a haplotype panel will require quadratic time complexity in terms of number of sequences. However, there are important distinct properties in haplotype panels compared to normal strings. The haplotype sequences are aligned, and also the amount of information in each location may vary due to abundance of locations with low minor allele frequencies. Furthermore, there is usually a correlation between the adjacent sites in the panel.

The Positional Burrows-Wheeler Transform (PBWT) [2] enables a fast and efficient method for haplotype data compression and searching for exact matches in a large panel of haplotype sequences linear to the number of sequences. The PBWT takes advantage of the properties of the haplotype panel and provides a linear search procedure and an efficient compression approach. PBWT is able to 1) find exact matches greater than a given length between all pairs of haplotypes in a panel, 2) find maximal substring matches of any haplotype in the panel and 3) search for a given query in a compressed haplotype panel. However, the presented algorithms by Durbin [2] only work for bi-allelic sites.

A bi-allelic site is a specific location in a chromosome that contains two observed values (alleles). Bi-allelic site can be represented as a binary sequence (containing 0’s and 1’s) where 0 denotes the reference allele (e.g. G) and 1 denotes the other observed allele (e.g. C). A multi-allelic site is a specific location in a chromosome that contains three or more alleles.

The most common type of genetic variation among individuals is Single Nucleotide Polymorphisms (SNP). SNPs are identified by the presence of a different DNA nucleotide from the reference allele at a specific location. Besides SNPs, structural variations are also common in the human genome. The structural variations deletions, insertions and duplications are referred as copy number polymorphisms (CNPs) and account for 4.8−9.5*%* of the human genome [3].

Multi-allelic SNPs have not been observed very frequently in the human genome. For example the fraction of tri-allelic SNPs in the human genome was estimated to be about 2% [4]. However, as more individual genomic data are becoming available, the fraction of estimated multi-allelic SNPs may change [5]. The number of multi-allelic sites among all variant sites is expected to increases non-linearly with the growing number of samples [5]. In a panel of 100,000 individuals, it is predicted that 6% of the variant sites would be multi-allelic [5].

Besides multi-allelic sites in SNPs, the possible alleles for CNP could be variable and in many cases there are more than two alleles. In one study nearly 1000 genes were identified with different segmental copy numbers ranging from 0 to 48 at 3 kb resolution [6]. The importance of multi-allelic CNPs cannot be replaced by only looking at their adjacent SNPs as their proxies. It has been shown that although bi-allelic CNPs show a strong correlation of copy number with flanking SNPs, a significant amount of multi-allelic CNPs residing in segmental duplications are not in linkage disequilibrium with nearby SNPs [7]. Hence, capturing the multi-allelic CNPs is also of interest. In addition, the complexity of the multiple copy numbers can be reduced by discretization of the numbers (e.g. integer values for the copy number or definition of ranges).

The number of multi-allelic sites could be much more than expected and sufficiently common in the human genome, so that the support for multi-allelic sites is necessary for any genomic tool or database [5]. Nevertheless, most genomic tools and repositories have ignored the multi-allelic sites. Those sites have either been discarded or converted into bi-allelic sites by assigning a possibly wrong allele to the sequences and ignoring the rare variants. PBWT algorithm is not an exception and it assumes that the haplotype panel is bi-allelic. However, as mentioned by Durbin [2], the bi-allelic version of PBWT can be modified to handle multi-allelic sites. In this paper, we present PBWT algorithms for multi-allelic sites (mPBWT). We assume that the number of possible alleles is limited and known. In the next section, we describe the construction of the data structure that enables a strong compression of the panel and also provides fast and efficient search approaches in terms of time and space, followed by two algorithms for finding long matches and longest matches between all the sequences within the panel. Finally, we describe the approach to compress the haplotype panel and recover the original panel for multi-allelic sites.

## Methods

### Prefix and divergence arrays

Durbin [2] presented a set of algorithms to construct positional prefix array data structures for bi-allelic sequences that can be used for time and space efficient sequence matching and compression of aligned sequences. The basic idea of the Positional Burrows-Wheeler Transform (PBWT) is to sort the sequences by their reversed prefixes. Given a sequence *s*=*s*_1_,*s*_2_,*s*_3_,..*s*_*N*_, the reversed prefix of *s* at a position *k* is *s*^*k*^=*s*_*k*−1_,*s*_*k*−2_,*s*_*k*−3_,…,*s*_1_. The first step is to create the positional prefix arrays in a given panel. At each position, the positional prefix array contains the indices of the sequences that are sorted by their reversed prefix order. For the multi-allelic version of the PBWT (mPBWT), we use similar notations as Durbin. Given a panel of *M* sequences and *N* variant sites with an alphabet of size *t*, we generate the positional prefix array *a*_*k*+1_ at the position *k*+1 by using the *a*_*k*_ and *x*_*k*_ values, where *x*_*k*_ contains the variant sites from *M* sequences at the position *k*. The prefix array *a*_*k*+1_ which is a permutation of the values {0..*M*−1} contains the indices of the sequences sorted by the reversed prefix at the position *k*.

For the bi-allelic version of PBWT, two auxiliary arrays *a* and *b* (for 0 and 1’s) were used to sort the sequences at each position efficiently. To extend it to t-allelic, we use a two dimensional array *a*. Algorithm 1 shows the procedure for computing the prefix array *a*_*k*+1_ from *a*_*k*_. The array *u* keeps track of the number for each allele at the position *k*.


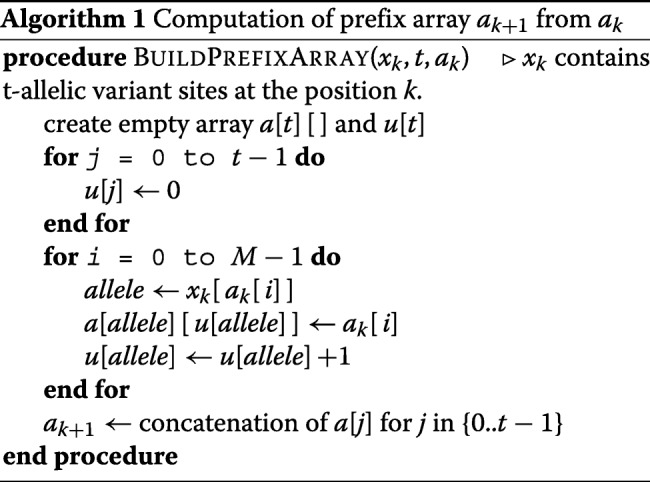


The time complexity of creating prefix arrays for multi-allelic sequences is similar to the bi-allelic. Counters for *t* possible alleles are initialized in the first loop and the arrays *a*[*j*] are concatenated at the end. The time complexity for creating the prefix arrays in a t-allelic panel will be *O*(*N*(*t*+*M*)). Assuming a fixed alphabet size, the complexity of creating prefix arrays will be *O*(*NM*).

After sorting the sequences by the reversed prefix order, each sequence will be adjacent to the sequence with the longest match. The divergence array *d*_*k*_ at the site *k* keeps tracks of the starting position of each match. *d*_*k*_[*i*] at each site *k* stores the position where the match between *x*[*a*_*k*_[*i*]] and *x*[*a*_*k*_[*i*−1]] begins. Divergence arrays for multi-allelic panels can also be constructed similar to the bi-allelic panel. The divergence array at the position *k* for the index *i* should be set to zero if *x*[ *a*_*k*_[ *i*]] ≠ *x*[ *a*_*k*_[ *i*−1]]. The bi-allelic version of the PBWT to construct the divergence array *d*_*k*+1_ from *d*_*k*_ uses two auxiliary variables *p* and *q* (for 0’s and 1’s). Here, we use an array *p*[] to update the divergence array at the indices where the first value of each allele appears in the sorted order. Table 1 summarizes the differences in data types between PBWT and mPBWT for computing prefix and divergence array. As shown in Algorithm 2, the procedure for creating prefix and divergence array contains two additional loops for *t* possible variant sites and also two concatenations at the end. The first loop only initializes the variables, and the second loop is for each sequence. As a results, the time complexity of computing prefix and divergence arrays will be *O*(*tNM*).

**Table 1:** Comparison of data types used in PBWT and mPBWT

	Prefix array		Divergence array	
PBWT	*a*[],*b*[]	*u*,*v*	*d*[],*e*[]	*p*,*q*
mPBWT	*a*[ *t*][]	*u*[*t*]	*d*[ *t*][]	*p*[ *t*]


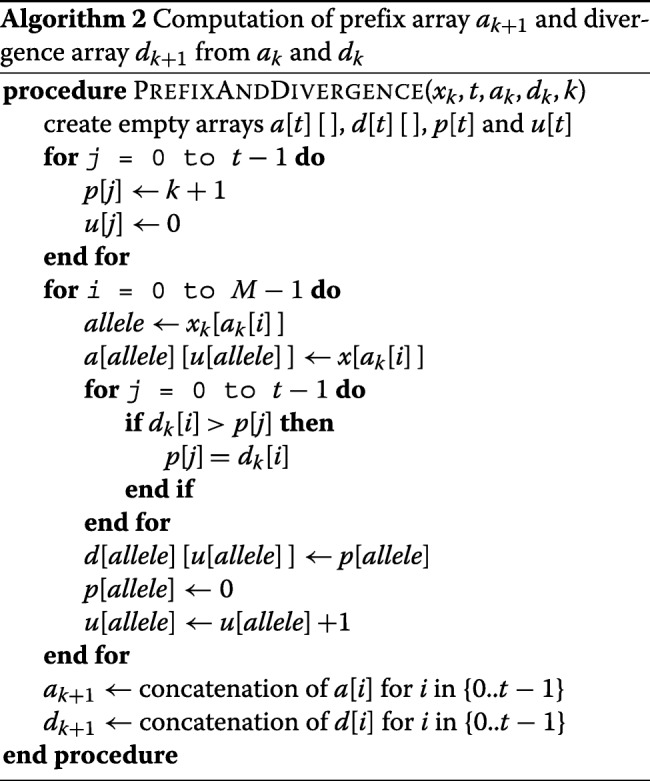


**Fig. 1:**
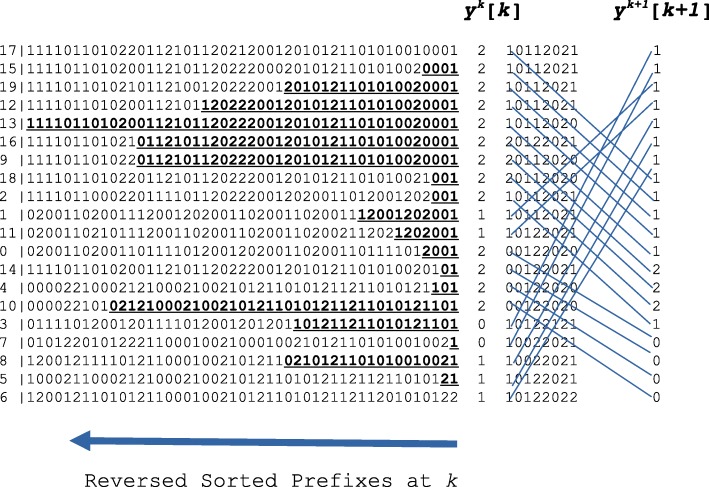
A multi-allelic panel (with three possible alleles) sorted based on the reversed prefix at the position *k* (not including *k*). The set of values at the position *k* is isolated and the right side shows how the order is derived at the position *k*+1. The array *y*[*k*] contains the sequences in their reversed prefix order

Figure 1 shows an example of a panel with three possible alleles. The array *y*^*k*^[ *k*] contains the values of *x*_*k*_ in their reversed prefix order: *y*^*k*^[ *k*] = *x*_*k*_[ *a*_*k*_]. The maximal match for each sequence is indicated by bold underline and the starting position of each bold underline equates the value of the divergence array for each sequence at the position *k*. And the prefix and divergence arrays will be updated when moving from position *k* to *k*+1.

### Finding all matches greater than a given length L

Sorting the sequences based on the reversed prefix order enables a fast search method to find all pairs of the sequences which share a long identical sequence. The bi-allelic version of the PBWT can find all matches greater than a given length in terms of number of sites in *O*(*max*(*NM*,*#matches*)). The algorithm provided by Durbin reports matches only if a mismatch occurs by definition. For example, two identical sequence will not be reported since the match will not terminate. While the majority of matches in a large panel will terminate at some point, the algorithm will fail in reporting some matches by the definition especially at the end of panel. Here, we extend the algorithm to handle multi-allelic data and also provide a solution to report all matches regardless of the terminating position. To count the matches greater than *L* which do not end at the panel, we handle the last variant site differently than the preceding sites.

Similar to bi-allelic search, we sweep though the panel and report the matches if a mismatch occurs. Algorithm 3 shows the algorithm for finding all matches greater than *L* sites that end at the position *k*. After sorting the sequences, all similar sequences will be placed in the same block separated by a sequence with *d*_*k*_[ *i*] >*k* −*L*. We iterate over the sequences at the position *k* and start reporting the matches if the starting index of the longest match for a sequence *i* is greater than *k* −*L* and we have already observed sequences with different alleles at the position *k*. The algorithm is different than the presented algorithm in [2] but similar to its implementation. The corresponding algorithm in [2] does not account for the starting position of the match, but in the implementation a slightly different approach had been used which was adapted in our algorithm. The additional array *m*[] for the multi-allelic version keeps track of occurrences of different alleles at the position *k*. A variable *report* triggers reporting the matches if a mismatch occurs which is detected when any two pairs of the entries of the array *m* have been set to *true*. To prevent comparing every two entries of the array *m*, we can simply iterate over all the entries of the *m* and increase a counter *s*. When the value of *s* is greater or equal to 2, we can start reporting the matches since a mismatch has occurred. At each block we report the matches from *i*_0_ to *i*, where *i*_0_ stores the last *i* where there was a block of matches greater than *L*. Finally, the block of matches may have not been separated by any sequence with *d*_*k*_[ *i*] >*k* −*L*. This case can happen for the last block. The final sequences will be reported at the end for the case that the block of similar sequences is not separated by a sequence *i*. Here, we omitted the details for simplicity but the same variables *s*, *report* and *i*_0_ are used to report the last block.


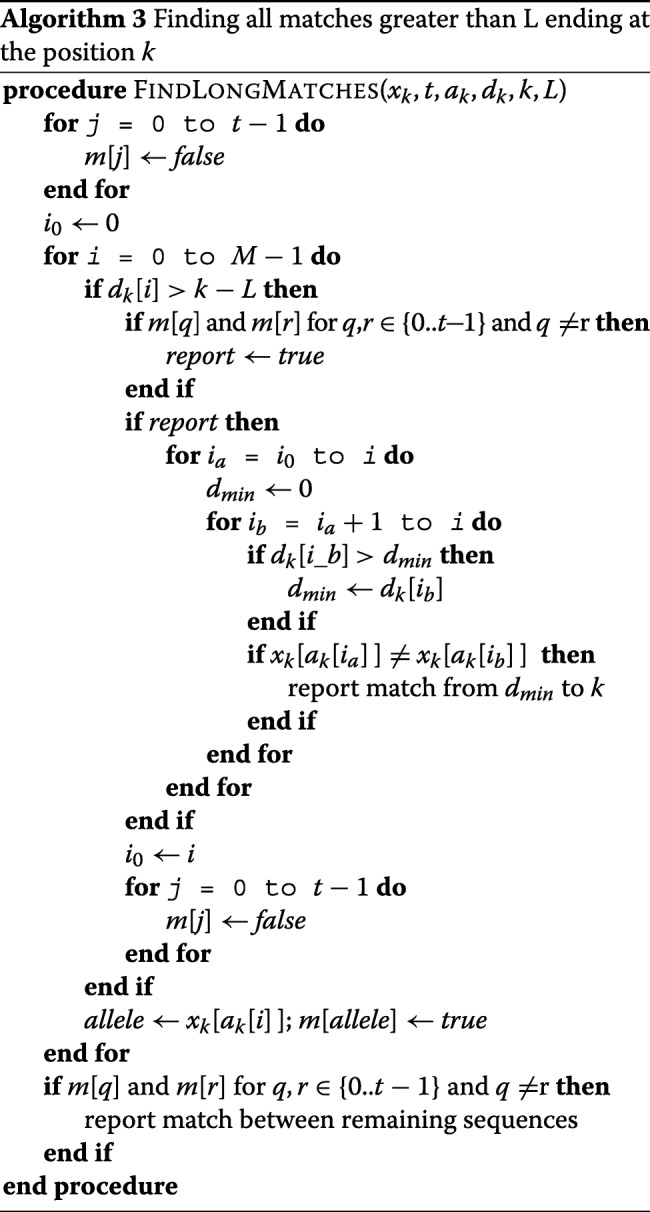


Algorithm 4 shows the routine for reporting all matches longer than *L* where *k* is the last site in the panel. Here, we search for matches greater than *L* − 1, hence the block of similar sequences is separated by a sequence with *d*_*k*_[*i*] >*k* −*L* + 1. Furthermore, we check the values of last site to make sure that the length is at least *L*. The reason is to cover the cases where a match occurs in [ *N*−*L*, *N*−1]. The time complexity of the algorithm for finding all matches greater than *L* will be *O*(*max*(*tNM*,*#matches*)), since for each site the array *m* with *t* elements is initialized and investigated.


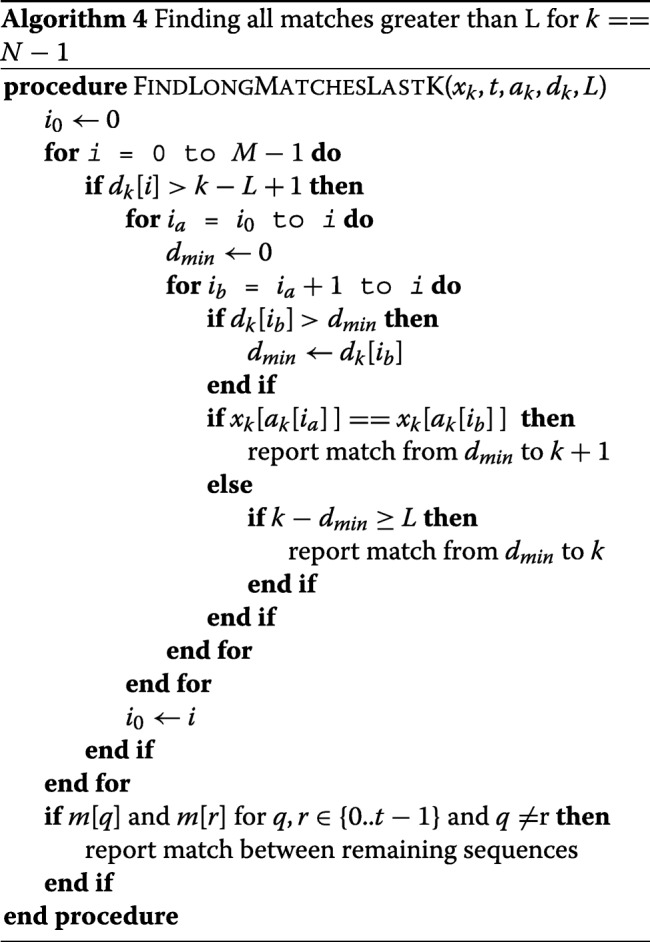


### Finding set maximal matches

A maximal match from a sequence *s* and a panel *X* at each position *k* is the longest match for *s*. The maximal match for *s* at the position *k* contains the sequence *x*_*j*_ if there is a match between *s* and the sequence *x*_*j*_ in the panel including [ *k*_1_,*k*), the match cannot be further extended, and there is no match with any other sequence *x*_*l*_ from the panel *X* including [ *k*^′^,*k*) where *k*^′^<*k*, or *k*^′^=*k*_1_ where *x*_*l*_[ *k*]=*s*[ *k*].

The algorithm for finding the set of maximal matches in multi-allelic version is similar to the bi-allelic version with the only difference in generating the divergence and prefix arrays. The positional prefix arrays and divergence array can be updated as we sweep through the panel as it was described before. The time complexity of generating divergence array is *O*(*tNM*). As a result, the time complexity for finding all set maximal matches will be *O*(*tNM*). The space complexity of the multi-allele version will be *O*(*M*+*t*) for a t-allelic panel. Furthermore, the algorithm provided by Durbin in [2], does not report a maximal match that does not end at the position *N*−1 by definition. Similar to long matches, we handle the case for the last *k* differently. Algorithm 5 shows the algorithm for finding set maximal matches at the position *k*, where *k*=*N*−1. The algorithm for finding set maximal matches at the position *k*≠*N*−1 is very similar to the bi-allelic version except the divergence and prefix array update, hence it is shown here.


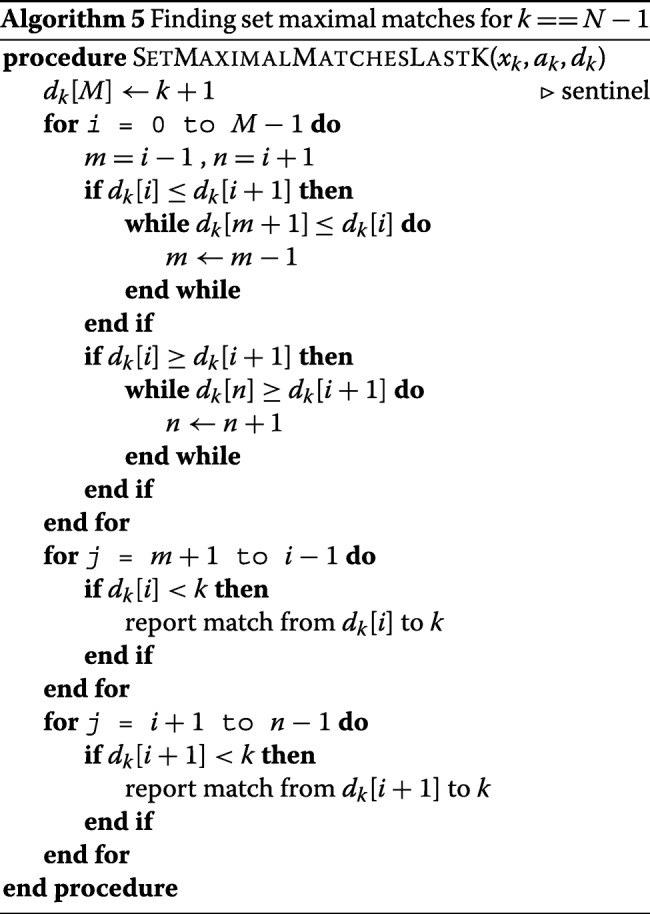


### Compression

Assuming the existence of linkage disequilibrium between the sites in a panel, sorting the sequences by reversed order will make the original panel more compressible. The reason is that if there is a local correlation between adjacent sites (linkage disequilibrium) and assuming a structure in the panel, then sequences with similar prefix values in the reversed prefix order would probably have the same value in the next variant site. As a result, the transformed panel will be more run-length compressible. The matrix *y*_*k*_[ *i*] and additional arrays which are similar to FM-index [8] in BWT [9] are compressed and stored. The array *y*_*k*_[ *i*] in PBWT contains the values for the sequences at the position *k* which are sorted by the reversed prefix order (not including *k*). In order to recover the original panel from the arrays *y*_*k*_ efficiently, we need to store the positional prefix arrays or additional matrices to compute the positional prefix arrays. The additional matrix *Occ* in PBWT corresponds to the matrix in BWT that stores the number of occurrences of each character *c* in the prefix *L*[ 1..*k*], where *L* denotes the last column in the lexicographically sorted rows of the rotations of the original sequence in BWT. The matrix *Occ*[ *l*][ *i*] contains the number of occurrences of the allele *l* up to the *i*-sequence in the prefix sorted order.

We define an array *c*(*t*) for each *k* which corresponds to the matrix in BWT that for each character *t*, stores the number of occurrences of lexically smaller characters. We also define an extension function *w*() similar to the bi-allelic version: *w*_*k*_(*i*,*l*)=*Occ*_*k*_[ *l*][ *i*]+*CC*(*l*−1), where *l* ∈ {0..*t*−1} and *CC* denotes the accumulative function of the number of alleles up to *l*−1 at the position *k*:

$$CC(l) = \sum\limits_{i=0}^{l} c(i)$$

One difference between the multi-allelic and bi-allelic version of PBWT for compression is that for the bi-allelic PBWT only the number of zeros for each site is stored, but here we have to store the number for all possible alleles except the allele *t*−1 in a t-allelic panel. The array *Occ* also replaces the arrays *u* and *v* in the bi-allelic version. Using the extension function, we can compute *a*_*k*+1_ from *a*_*k*_ or vice versa: *a*_*k*+1_[*w*_*k*_(*i*,*y*_*i*_[*k*])]=*a*_*k*_[*i*]. As a result, we only need to store a subset of prefix arrays and we can compute the remaining arrays efficiently. Algorithm 6 shows the computation of FM-index for the position *k*+1 as we move from *k* to *k*+1. Please note that for the first variant site *k*=0: the PBWT array *y* is *y*_*k*_[*i*]=*x*_*k*_[*i*] and the positional prefix array is *a*_*k*_[*i*]=*i*. Once the arrays *y* is computed, the same compression algorithm for bi-allelic can be used to compress the data. The matrix *Occ* is also run-length compressible. In this work, we did not introduce the search for a given query sequence in a compressed panel. However, to search for a query in a panel, the divergence arrays need to be stored as well. The compression of divergence arrays will be similar to the bi-allelic version.


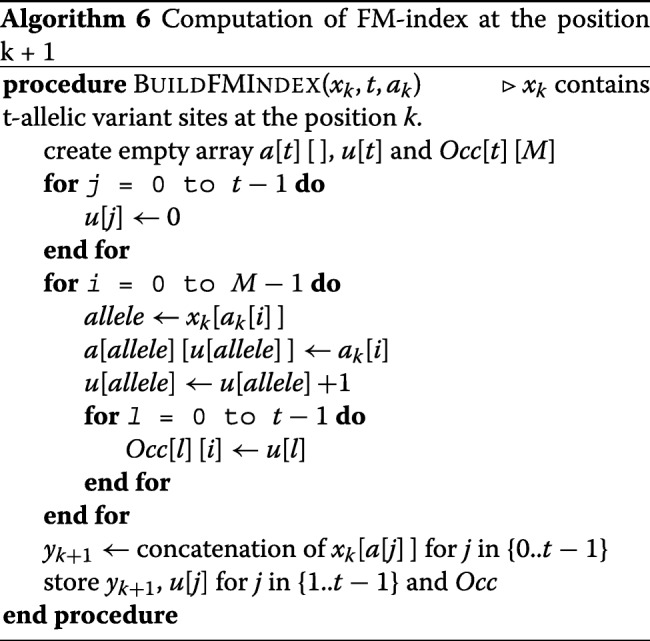


## Results and discussion

Table 2 shows the time complexity differences between PBWT and mPBWT.The time complexity of PBWT for multi-allelic version increases by a factor of *t*, where *t* denotes the possible alleles in each site. The space complexity for searching the long matches or set maximal matches is *O*(*M*+*t*) compared to the *O*(*M*), where *M* denotes the number of haplotypes.

**Table 2:** Time complexity comparison between PBWT and mPBWT

	PBWT	mPBWT
Prefix array	*O*(*NM*)	*O*(*N*(*t*+*M*))
Divergence array	*O*(*NM*)	*O*(*tNM*)
Long matches	*O*(*NM*,*#matches*)	*O*(*tNM*,*#matches*)
Set max matches	*O*(*NM*)	*O*(*tNM*)

We tested our implementation with a panel of bi-allelic data with 4000 haplotypes and 94992 variant sites, generated by MaCS simulator [10]. The time for computing all long matches in our implementation was similar to the original PBWT for bi-allelic data. We also transformed the panel into tri-allelic and 10-allelic by converting the 0’s or 1’s in 30% of the variant sites. Table 3 shows the running time values for different cases. In general, the running time for finding matches is mainly impacted by the number of matches and not the number of possible alleles. Please note that the number of short matches is significantly higher in a real haplotype panel. Table 4 shows the running time for finding set maximal matches with the increasing number of haplotypes. As shown in the table, the running time increases linearly with the increasing number of sequences.

**Table 3:** Time to find matches in seconds using different minimum lengths in terms of the number of sites

Minimum length	2000	1000	500
PBWT	5.496 s	7.443 s	37.999 s
mPBWT	5.528 s	7.314 s	36.796 s
mPBWT (tri-allelic)	6.103 s	8.329 s	38.247 s
mPBWT (t = 10)	13.740	14.534	38.742 s

**Table 4:** Time to find set maximal matches in seconds using different number of haplotypes

Number of haplotypes	1000	2000	4000
PBWT	4.141 s	7.873 s	15.053 s
mPBWT	3.756 s	7.882 s	14.185 s
mPBWT (tri-allelic)	4.213 s	8.780 s	15.602 s
mPBWT (t = 10)	4.219 s	10.190 s	19.533 s

All of the presented algorithms cannot handle genotype error as the original PBWT. Genotype errors often interrupt long matches into multiple short fragments. One solution to handle genotype errors is to search for short seed matches. However, this approach would require an appropriate mechanism to identify seed length since the number of short seed matches may be very high in a genotype panel. Another approach is to consider a top-down approach as was used in [11]. In this approach, multiple PBWTs are generated on random projections of the original panels. Basically, we search for exact matches in multiple sub-samples of the panel. For the estimation of the sub-sampling rate, a multinomial distribution can be considered instead of the binomial distribution. The provided algorithms also do not account for missing data. However, the missing values can be coded as an integer value and the panel can be compressed even with its missing values. mPBWT does not provide a direct solution to handle the missing values in a panel. However, it could make it easier to implement heuristics based on certain assumptions. If we define a new allele for missing data, in the sorted prefix order the block with the missing value at their last prefix position would be at the bottom. Assume that there are no consecutive missing values, we can update the missing value to generate longer matches for each sequence considering the length of the match by including the immediately preceding and following sites of the missing site.

## Conclusion

In this paper, we presented a series of algorithms to generate positional prefix arrays and divergence arrays for multi-allelic data based on the original PBWT for bi-allelic data. Furthermore, we presented algorithms for finding long matches and set-maximal matches in the panel. The multi-allelic PBWT is time and space efficient and also would be comparable to the bi-allelic version as long as the number of allele sites is not very high. Particularly, the time complexity of mPBWT is increased by a factor of *t*, where *t* denotes the number of possible alleles.

In our implementation, we assumed a constant *t* for the entire panel. However, the number of possible alleles across a chromosome may vary. As we iterate over the sites, we can use different values for *t* independently. This would be useful, especially if the number of sites with many possible alleles is low. As a result, searching for maximal matches or long matches would be more efficient and the average memory consumption would be lower as we sweep through the panel to report the matches and update the prefix and divergence arrays.
